# Burnout and team functioning in pediatric pain care: A cross-sectional survey of multidisciplinary providers

**DOI:** 10.1016/j.jpain.2026.106206

**Published:** 2026-02-05

**Authors:** Courtney W. Hess, Katrina Huft, Emma Francesca Gaydos, Monserrat Hernandez Escobar, Lita Moua, Dionne Chen, Ashley McDonnell, Laura E. Simons

**Affiliations:** a Stanford University School of Medicine, United States; b Duke University School of Medicine, United States; c Stanford University, United States

**Keywords:** Burnout, Teamwork, Pediatric Pain, Chronic Pain, Integrated Care

## Abstract

Burnout is a concern among healthcare providers, linked to suboptimal patient care. Working as part of an integrated team has been identified as a mitigator and exacerbator of burnout, however, this relationship is not well understood in pediatric chronic pain care. This study assessed levels of burnout and perceptions of teamwork in a multidisciplinary group of pediatric chronic pain providers. A cross-sectional survey consisting of the Copenhagen Burnout Inventory and team functioning measures (i.e., communication/information sharing, team support, team effectiveness) was completed by N=195 providers. Pearson correlations were conducted to evaluate the association between burnout and teamwork. A K-means cluster analysis was used to group providers according to their level of burnout, and groupwise comparisons were conducted to examine differences in team functioning perceptions across established groups. Rates of personal (M=39.91, SD=16.97) and work-related (M=39.67, SD=17.75) burnout exceeded established norms. Team functioning ratings were generally positive; however, variability existed with poorer ratings in areas such as team coordination and productivity. Increased burnout was associated with poorer perceptions of team effectiveness and higher perceived room for improvement. K-means clustering identified three distinct provider groups: high, moderate, and low burnout, with significant differences in perceived team functioning across these groups. Elevated rates of burnout exist among multidisciplinary pediatric pain providers and are related to perceptions of team functioning. Research should explore directionality and causality of this relationship as well as provider experiences to support development of interventions to address team functioning and burnout and thus improve patient care.

## 1. Introduction

Burnout is increasingly prevalent among healthcare providers^1-3^ with pain physicians reporting some of the highest rates in medicine.^4,5^ All healthcare providers are at risk of burnout, however, elevated rates in pain care have been attributed to increased diagnostic uncertainty for patients, families, and providers, suboptimal outcomes for existing interventions, high rates of psychological comorbidities among youth with pain, and elevated emotional stress faced by clinicians.^6,7^ Additionally, in chronic pain care, where integrated or team approaches to assessment and treatment have been identified as the gold standard,^8-10^ colleague interactions have been identified as both a mitigating and contributing factor to provider burnout.^11^ That is, operating as part of a team may have the potential to mitigate against burnout, but may also contribute to burnout^11^ as a team approach may reduce individual provider burden and offer social support, but can also be difficult to execute requiring additional time, skills, and energy from providers.^12^

Unaddressed burnout has been linked to decrements in patient care including patient safety and care quality,^13^ medical errors,^3,14^ and negative patient-provider interactions.^3,15,16^ Burnout is also associated with provider departures from healthcare,^3,17,18^ exacerbating access difficulties and increasing the burden on remaining healthcare providers.^3^ As such, addressing burnout among pediatric pain care providers is essential to patient care, yet a dearth of research exists and studies are most often limited to a single discipline (e.g., medicine, nursing)^5,19^ and do not reflect the nature of multidisciplinary pain care. Moreover, existing interventions to mitigate burnout also largely target individual provider factors (e.g., mindfulness-based interventions, self-awareness training, coaching) and to a much lesser extent organizational factors (e.g., task restructuring, job crafting).^20^ These interventions have demonstrated positive impact, however, limitations exist.^21^ Individual interventions are often underutilized and have short lasting effect and organizational interventions can be difficult to actualize or implement.^22^ There is a limited number of interventions that have targeted interpersonal factors (e.g., improving communication between team members), however, interventions to date have demonstrated promise.^21,23^ Targeting burnout at the interpersonal level may offer a path forward that matches current approaches to pain care while also providing more robust, long-lasting, impact with increased implementation feasibility. However, the relationship between interpersonal factors (e.g., team functioning factors) and burnout in pediatric chronic pain care is not yet well understood.

As such, the current study examined burnout and perceptions of team functioning in a multidisciplinary sample of pediatric chronic pain providers. The overarching goal of this work is to evaluate the relationship between provider perceptions of team functioning and their experiences of burnout in an effort to identify novel pathways through which burnout may be mitigated (i.e., addressing suboptimal team functioning). As a result, this work may alleviate known consequences of healthcare provider burnout, including decrements to patient care.^3,13^

To achieve this aim, we deployed an international cross-sectional survey to a multidisciplinary group of pediatric pain providers who were working as part of an integrated pain care team. We hypothesized that: (a) rates of burnout would be elevated among all pediatric pain providers compared to established norms among service work providers,^24^ and (b) provider perceptions of team functioning would be inversely related to their levels of burnout, such that as perceptions of team functioning decrease, levels of burnout will increase.

## 2. Materials and methods

### 2.1. Design

The current study employed a cross-sectional design. Survey data were collected from individual pain care providers working in multidisciplinary pain care clinics across the world.

### 2.2. Ethical considerations

The study was granted ethical approval from the Institutional Review Board of Stanford University (eProtocol#67279). To protect the identities of participants, surveys were able to be completed anonymously, and clinicians were not asked to provide identifying information (e.g., name of their pain clinic). Survey data were collected and managed using REDCap electronic data capture tools hosted at Stanford University.^25,26^ Following completion of data collection, data were downloaded and stored in password-protected, encrypted, servers at Stanford University. Only study team members had access to the survey data. Before initiating the survey, participants were informed of the topic areas represented in the survey (burnout, perceptions of teamwork). Participants were also informed that no identifying information would be collected so that all responses could remain anonymous. Participants completed the clinical characteristics information first before answering questions about burnout or teamwork to provide assurance that no identifying information would be attached to their responses. Consent was implied through completion of the survey, and no consent signatures were collected to preserve anonymity.

### 2.3. Patient involvement

A patient partner (AM) was involved in the design and conduct of this research alongside healthcare provider partners and the BETTER panel; a patient partner panel embedded within the biobehavioral pediatric pain lab. In the early stages of study design, the lead author (CWH) met with the BETTER panel to evaluate the relevance and impact of examining provider burnout in pain care teams and elicited feedback from youth with lived experience regarding the study purpose. Across the course of the study including conceptualization, design, data analysis, and interpretation co-author (AM) who is a young adult with lived experience of pediatric chronic pain contributed their expertise. Involvement was focused on ensuring that the research questions were important to people with lived experience (study conceptualization and design) and considered the way in which these concerns impact patients and patient care delivery (interpretation and discussion). Additionally, in the formation of the survey multiple providers including physiotherapists, physicians, and psychologists reviewed the survey to ensure the questions were clear, relevant, and sensitive to the diverse roles on a pediatric pain team.

### 2.4. Clinical setting

The survey was directed toward providers working within integrated or multidisciplinary pain teams. The clinical settings varied across providers including inpatient pain service teams, outpatient pain clinics, and intensive interdisciplinary pain treatment settings. All providers, regardless of setting, were asked to indicate whether they were part of a multidisciplinary team of pain providers. Multidisciplinary pain teams consist of two or more providers from diverse backgrounds that work in a coordinated fashion to assess and treat pain from a biopsychosocial lens. Composition of teams varies, but can include physicians (e.g., anesthesiologists, physical medicine and rehabilitation, pediatricians), psychologists, nurses and nurse practitioners, physical and occupational therapists, social workers, and nutritionists.

### 2.5. Procedure

Recruitment of providers occurred in two ways. First, a list of pediatric pain clinics (N=85) was created using the list of pediatric pain management clinics compiled yearly by the International Association for the Study of Pain (IASP), pain in childhood, special interest group. The identified director of each clinic was contacted directly by the first and senior authors and asked to complete the survey and share the survey link with members of their pain team(s). Second, the survey link was distributed via professional listservs, including pain-specific listservs, pediatric psychology, and physiotherapy listservs to elicit engagement from a variety of professionals in pediatric pain care. The email and embedded link allowed providers to self-initiate the survey at their discretion. The survey included four measures consisting of 49 total items and a single open-text item. The survey took an average of 15–20 min to complete. Recruitment started in July of 2023 and was completed in January of 2024.

### 2.6. Measures

#### 2.6.1. Individual and team characteristics

Providers were asked to provide demographic data (e.g., age, gender identity, racial identity), characteristics of their professional identity (e. g., professional domain, training, years of experience), and characteristics of their clinic and clinical role (e.g., hours spent in direct care, type of pediatric pain care team, clinical setting).

#### 2.6.2. Symptoms of burnout

To understand individual provider experiences of burnout, participants completed the **Copenhagen Burnout Inventory** (CBI).^24^ The CBI is a 19-item self-report measure of burnout. It contains three sub-scales that include work-related burnout, personal burnout, and client-related burnout. The CBI was validated in a cohort of employees working in the human service sector in Denmark as part of a larger longitudinal study on burnout and motivation in Denmark (i.e., Project on Burnout Motivation and Job Satisfaction; PUMA).^27^ Participants respond to each item using a 5-point Likert-type scale ranging from never/almost never (score of 0) to always (score of 100). Each CBI item is scored on a 0–100 scale in increments of 25. Items are averaged in each subscale to give a subscale burnout score, with lower scores representing less burnout in the respective area (personal, work-related, client-related). Average CBI ratings for each subscale have been published for the total cohort as well as for each subgroup of service workers. The CBI was selected for the current project as it is freely available and would allow for reproducibility and more expansive recruitment efforts. In addition, the CBI’s focus on sources of burnout allowed for differentiation between factors related to the individual, the patient-provider relationship, and the provider’s work and job more broadly. Cronbach’s alpha was high across all three subscales. Personal burnout consisted of 6 items and Cronbach’s alpha was 0.88. Work-related burnout consisted of 7 items and Cronbach’s alpha was 0.87 in this sample. Client-related burnout consisted of 6 items and alpha for this sample was 0.86. Total burnout was the sum of all 19 items and Cronbach’s alpha was 0.93.

#### 2.6.3. Team functioning

To assess teamwork in pediatric pain care teams, questions regarding team characteristics as well as two measures of team functioning were employed. **Team characteristics** assessed were: 1) types of providers involved in their team (e.g., nursing, physicians, physiotherapist), 2) the number of years they had been part of their team, and 3) whether any significant changes had occurred in their team in the last year (e.g., new leadership, restructuring of the team). Additionally, prior to starting the team functioning survey, authors acknowledged that providers may be part of multiple pain teams and prompted providers to indicate the team they were referring to as they responded to the questions. To ensure all providers were actively part of a multidisciplinary team, response options included (1) I do not currently work as part of a multidisciplinary team, (2) outpatient multidisciplinary pain treatment, and (3) intensive interdisciplinary pain treatment. If a provider responded with a 1 the survey ended and they were not included in analyses. Providers also completed a subset (8 items) of adapted items from the **Communication and Sharing of Information Scale** (CSI scale). The CSI scale is a 13-item self-report measure focused on perceptions of communication with medical staff as well as information sharing between medical staff.^28^ Items were adapted to reflect the multidisciplinary nature of pain teams (e.g., changing “*physicians and nurses* share medical information they received or delivered to the patient” to “*providers on my pain team* share medical information they received or delivered to the patient”). A subset of the measure was completed as some items were deemed not relevant to the current study (e.g.,” it’s easy to ask nurse assistants for advice in the unit”) and other items were deemed redundant after adaptations to language were made. Participants were asked to respond to a series of questions using a 4-point Likert scale ranging from never (score of 1) to always (score of 4) with higher scores indicating better perception of communication and information sharing among the team. Cronbach’s alpha was high for the measure (0.94). The final measure completed by providers was the **Team Functioning Scale**,^29^ a 42-item measure that asked providers to rate their team members on their team support and several aspects of team effectiveness including but not limited to leadership, cohesion, shared decision making, and problem solving. For the current study, we selected subscales focused on team support and team effectiveness. Team support is comprised of 9 items (e. g., the members of my team spend very little time encouraging each other, team members take a personal interest in other members). Participants rated team support statements on a 6-point Likert-type scale ranging from true (score of 1) to false (score of 6) with 6 items being reverse scored so that higher scores indicated more team support. Cronbach’s alpha for the team support subscale was high (0.92) for this subscale. Team effectiveness consists of 17 items (e.g., overcoming misunderstandings among team members, coordinating team activities, achieving treatment goals). Participants rated facets of teamwork using a 7-point scale ranging from not very effective (score of 1) to very effective (score of 7). Responses were analyzed individually and averaged across all 17 items to get a team effectiveness score where higher scores indicated higher perceived effectiveness. Cronbach’s alpha was high (0.96) for this subscale. Participants were also asked to rate their perceived room for improvement on the 17 items ranging from no room for improvement (score of 1) to a lot of room for improvement (score of 7). Responses were analyzed individually and averaged across all 17 items to get a room for improvement score where higher scores indicated more room for improvement on the team. Cronbach’s alpha was high (0.95) for this subscale.

### 2.7. Data analysis

All statistical analyses were performed using R software (version 4.4.1).^30^ First, data were assessed for missingness. Following recommendations from Newman et al., data were assessed for level of missingness (item, construct, person) and type of missingness (completely at random, at random, not at random), and treated accordingly.^31^ We used the maximum available observations for each construct in the analyses. Given, the minimal amount of missing data for the burnout measure (0.2%) and as all missing data were item level and considered missing completely at random (i.e., less than 5% missing, no correlation with other variables), missing data were treated with item level multiple imputation using the MICE package in R.^32^ For the team functioning measures, most missing data were at the construct level (i.e., providers ended the survey early and did not answer any team functioning questions). As such, we did not use imputation and instead used maximum available observations, excluding participants with missing data. Necessary items were reverse coded to allow for sum scores to be tallied. Sum scores for all subscales were created including personal burnout, work-related burnout, client-related burnout, total burnout, total communication and information sharing, team effectiveness, and team room for improvement. Additionally based on professional role, respondents were categorized according to their discipline which included medicine, behavioral health, rehabilitation, nursing, and other. Behavioral health included social workers, psychologists, and counselors. The rehabilitation domain included physical, occupational, and recreational therapists. Lastly, the “other” domain consisted of massage therapists, child life specialists, music therapists, school liaisons, and program coordinators.

#### 2.7.1. Demographic and clinical characteristics in relation to provider burnout & team functioning

Descriptive statistics (mean, standard deviation, range, frequencies) were calculated to describe burnout levels and perceptions of teamwork across pain providers and describe the clinical characteristics of providers in this sample including clinical setting, hours spent in clinical care, number of patients seen per week, hours spent in clinical coordination, and types of coordination activities. Mean burnout scores were compared to established norms published in the CBI validation study.^24^ Although authors of the CBI did publish mean burnout scores for a variety of health service sector roles, not all domains in the current study were represented in the subgroup means. As such, we have presented the range and mean scores for each subscale with scores greater than the published *mean* being described as higher than established norms.

Groupwise comparisons were also conducted to evaluate differences in clinical characteristics, burnout, and perceptions of teamwork across salient demographic (age, gender), and clinical characteristics (e.g., professional domain, years of experience) across provider domains (e.g., medicine, psychology).

#### 2.7.2. Relationship between burnout and team functioning

To evaluate whether a relationship exists between perceptions of teamwork and levels of provider burnout, scatter plots were created, and Spearman Rank Correlations calculated given that the data demonstrated significant skew. Correlations were calculated between burnout subscales (including personal, client-related, and work-related burnout) and provider perceptions of teamwork (including perceived quality of communication, team support, and perceived team effectiveness and room for improvement). Alpha was set to level.05.

#### 2.7.3. Differences in team functioning across providers with different levels of burnout

As significant relationships emerged between aspects of burnout and team functioning, we further explored this relationship to evaluate whether there were significant differences in team functioning between providers who were experiencing different levels of burnout. K-means cluster analysis and groupwise comparisons were used to examine these differences. To separate providers into meaningful groups according to their level of burnout, a K-means cluster analysis was used.^33^ K-means clustering is an iterative unsupervised machine learning technique designed to separate data into K groups based on an identified parameter or parameters, where K is the pre-determined number of groups as decided by the researcher. In the current study, the parameters used to partition providers into groups were personal, work-related, and client-related burnout scores. To establish the optimal number of clusters (i.e., number of centroids) we followed established practices including the elbow method, gap statistic, and average silhouette method.^34^ Once the optimal number of centroids was determined, the K-means clustering algorithm worked in an iterative process to create groups that maximized similarity of scores within the group while optimizing difference (i.e., Euclidean distance) between groups.^35^ The iterative process ended when convergence was achieved. Convergence was defined as two successive iterations that result in less than or equal to 0.01 movement in centroids. To examine differences in team functioning between the groups of providers, groupwise comparisons were conducted.

## 3. Results

### 3.1. Sample characteristics

A total of N=254 providers opened the survey with n=224 completing items across at least one construct (demographic data, burnout inventory, team functioning measures). Of the n=224 providers who provided responses, we removed providers who either responded “I am not currently working on an integrated care team” n=15 or did not respond (n=14) to our question about the type of team they worked on. After removing those providers, a total sample of N=195 providers completed items across all measures and reported that they were part of an integrated pain care team. Providers varied across professional domains including psychology, social work, medicine, nursing, physical and occupational therapy, recreational therapy, and teaching. Providers also worked across different clinical contexts including outpatient multidisciplinary pain clinics, intensive interdisciplinary pain treatment programs, and inpatient pain services. Most providers reported being between 31 and 50 years of age (n=120; 62%) and had between <1–10 years of experience (n=118; 61%). Clinical commitment for providers also varied from a minimum of 1–10 h spent in clinical care (n=67; 35%) to a max of over 50 h spent in clinical care (n=5; 3%). A full description of the sample including demographic and clinical characteristics is provided in Table 1. In addition, a full description of the frequency and types of clinical coordination activities reported by providers is summarized in supplementary table 1.

### 3.2. Prevalence of burnout

Within the current sample, the highest reported burnout subscale was personal burnout (M=39.91±16.97) followed by work-related burnout (M= 39.67±17.75), and then client-related burnout (M=25.62±18.13). Compared to averages for professionals in the human service sector published by Kristensen et al. in the CBI validation study,^24^ mean scores for providers in the current sample were, on average, higher for personal and work-related burnout and lower for client-related burnout. Although mean burnout scores varied across professional domains, no significant differences in burnout scores emerged. Within the current sample, the mean scores for personal burnout were the largest among nursing (M=42.26 ± 20.51), followed by rehabilitation providers (M=42.00 ± 16.18). For work-related burnout, rehabilitation providers had the largest mean score (M=43.37 ± 18.92) followed by providers who fell into the “other” domain including massage therapists, child life specialists, music therapists, school liaisons, and program coordinators. The largest mean score for client-related burnout was among rehabilitation providers (M=29.70 ± 20.00) followed by medical providers (M=27.08 ± 18.21). Full description of burnout scores across professional domains is reported in Table 2.

### 3.3. Teamwork characteristics

Provider perceptions of teamwork were negatively skewed across all team functioning scales, indicating that most respondents reported more positive ratings of their teams’ functioning. Providers within the medical domain reported the most positive perceptions of team communication and information sharing (M=27.21 ± 4.64), team effectiveness (M=5.18 ± 1.06) and felt team support (M=45.51 ± 7.53). Although mean scores for these providers were the highest, there were no significant differences between professional domains for any of the facets of team functioning. Regarding team effectiveness, respondents rated their team highest in their ability to be ethical in their work, establish treatment goals, and work together as a team. In contrast, respondents rated their team lowest in their ability to use team meetings productively, coordinate team activities, try innovative strategies, and overcome team misunderstandings. Consistent with ratings of team effectiveness, providers reported the most room for improvement in their team’s ability to use meetings productively and try innovative strategies. However, differing from team effectiveness, providers reported problem solving complex or challenging patients as the third highest area with room to improve. Total team functioning scores across professional domains is provided in Table 3 and mean ratings of team effectiveness and room for improvement are provided in Table 4.

### 3.4. Relationship between teamwork and burnout

**Fig. 1.**
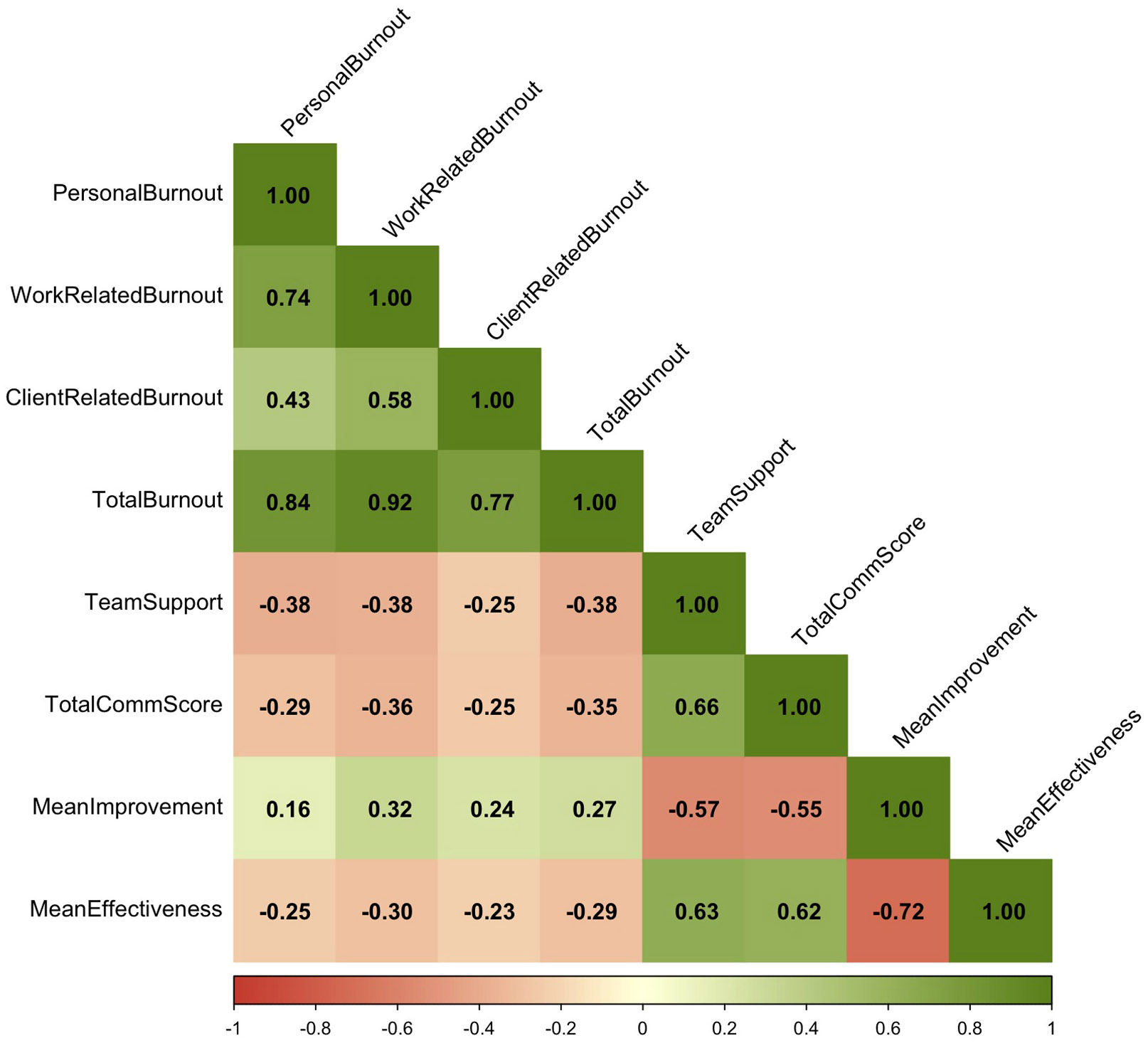
Spearman Rank Order Correlation Matrix (Teamwork Factors & Burnout). Colors denote a positive (green) and negative (red) correlation with more pale colors indicating weaker correlations and more intense colors representing stronger correlations.

As no significant differences in burnout or reported team functioning emerged across professional roles or clinical contexts (outpatient, intensive rehabilitation), the whole sample was used for correlation and cluster analyses. Results of the Spearman rank order correlation analyses revealed significant correlations between provider experiences of burnout and their perceptions of team functioning. That is, there were significant correlations between personal, work-related, and client-related burnout and all aspects of team functioning including perceived room for improvement, current team effectiveness, communication and information sharing, and felt team support. A full correlation matrix is provided in Figure 1. Relationships between constructs were in the anticipated direction such that as levels of burnout increased, perceptions of team functioning worsened. This relationship was consistent across all subscales of burnout. Regarding perceived room for improvement, the relationship was flipped such that as provider burnout levels increased so did their perceived room for improvement within their team.

### 3.5. Distinct groups by burnout level

**Fig. 2.**
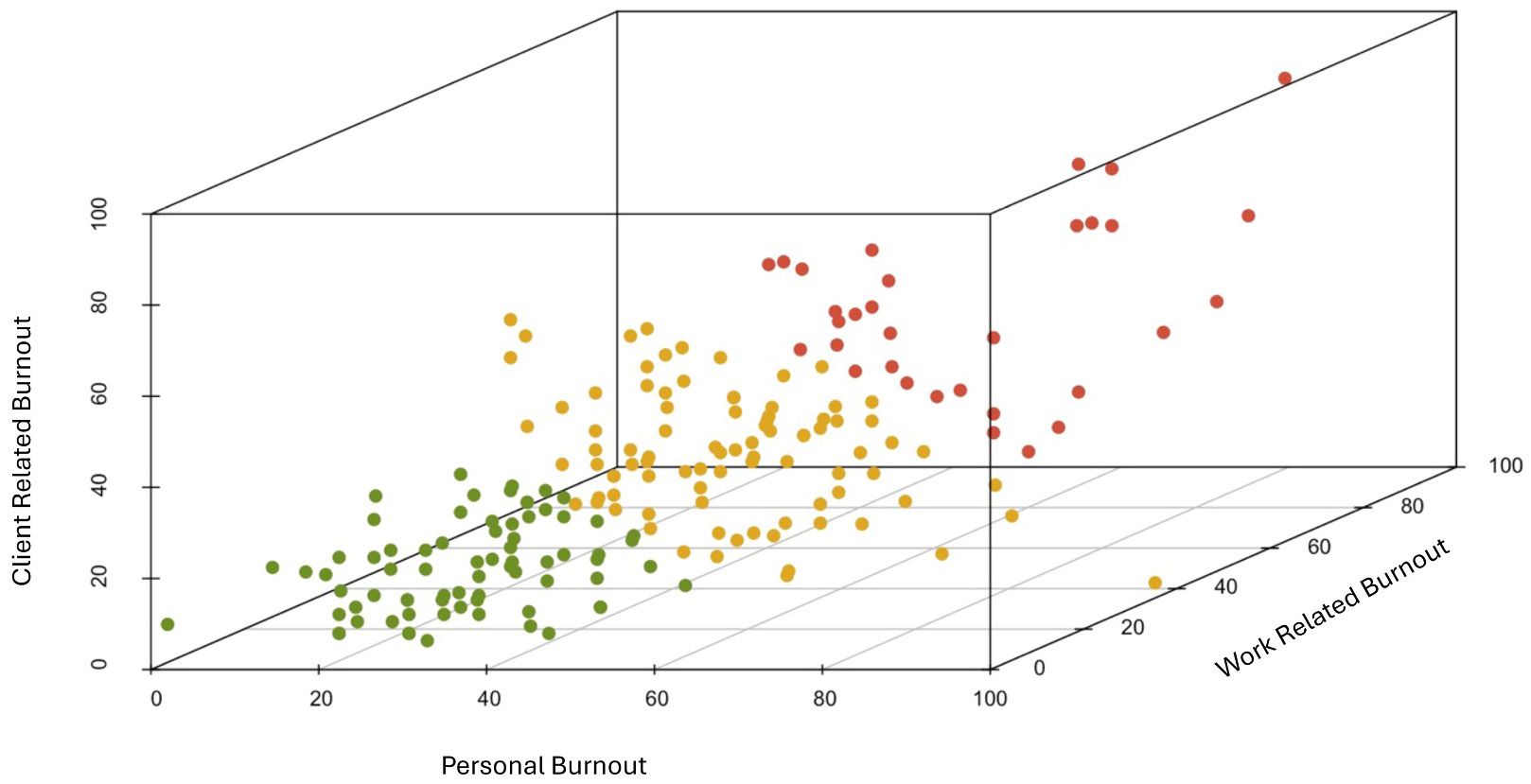
K-means Cluster Analysis Plot. Colored dots indicate the final categorization of the provider such that green is low burnout, orange/yellow is moderate burnout, and red is elevated/high burnout.

A cluster analysis was conducted to assess whether patterns or distinct groups existed among respondents based on their levels of burnout. Results of the elbow method indicated a three or four-cluster solution to be optimal, while the gap statistic and silhouette method indicated a two or three group solution. Taken together, we selected the three-cluster solution as it demonstrated statistical superiority including good separation between groups (i.e., betweenness) and good compactness within groups (i.e., withinness). Results of the analysis identified distinct groups of providers, representing providers with *high* levels of burnout (N=32), providers with *low* levels of burnout (N=74), and providers with moderate levels of burnout (i.e., elevated symptoms) placing them potentially *at-risk* for burnout (N=89). Cluster center means across personal, work-related, and client related burnout were statistically significant, indicating that the clustering analysis did separate meaningfully according to burnout severity (see Figure 2 for scatter plot of three groups across three dimensions of burnout). For personal burnout, providers in the high burnout group reported a mean score of 61.33 ± 11.48, whereas the moderate burnout group reported mean scores of 44.43 ± 12.31 and the low burnout group reported a mean score of 25.23 ± 8.83. For work-related burnout, providers in the high burnout group reported a mean score of 65.85 ± 12.83 while those in moderate burnout group reported 44.06 ± 8.90 and the low burnout group reported a mean score of 23.07 ± 8.30. Finally, for client-related burnout, providers in the high burnout group had an average score of 49.74 ± 17.42 compared to the moderate burnout group which had a mean burnout score of 28.14 ± 13.62 and the low burnout group who had a mean score of 12.16 ± 8.63. Cluster centers across burnout scores are reported in Table 5.

As three distinct groups had been clearly defined, groupwise comparisons for unequal sample sizes were conducted to evaluate whether differences in clinician characteristics or perceptions of team functioning differed between the groups. Groupwise comparisons did not reveal any significant differences in demographic or clinical characteristics such as provider professional domain, number of patients seen per week, hours spent in clinical care each week, or years of experience. Significant differences did emerge, however, between the groups related to their perceptions of team functioning such that across all facets of team functioning providers in the high burnout group rated their team more poorly than providers in the moderate and low burnout groups and those in the moderate group rated their team more poorly than providers in the low burnout group. Full results of the groupwise comparisons across the established groups are provided in Table 6.

## 4. Discussion

The purpose of the current study was to examine levels of burnout and perceptions of team functioning from providers working in integrated pediatric pain care teams. Previous research has often been restricted to a single professional domain (e.g., nursing, medicine) and has not focused on providers who are integrated into multi-disciplinary teams, which could be a buffer but may also exacerbate burnout due to the increased time and emotional demands of working as part of a team. We assessed the relationship between provider levels of burnout and their perceptions of their teams’ functioning. Findings indicate that burnout is a concern among providers who work within pediatric pain care teams. Additionally, results suggest that provider burnout and perceptions of team functioning are related such that providers with higher levels of burnout reported worse perceptions of their team across several facets of team functioning (e.g., communication, felt support, effectiveness).

### 4.1. Burnout in pediatric pain care teams

The current findings indicate that providers across several professional domains in pain care experience burnout, reinforcing the growing concern in healthcare related to provider burnout and associated concern for practitioners opting to leave the healthcare field.^36-39^ While several mean burnout scores were elevated, individual responses varied such that some reported significantly elevated levels of burnout while others reported minimal to no burnout. To this end findings highlight the individual nature of burnout as burnout did not differ across professional domains. Consistent with existing literature, physicians in the current study reported elevated levels of burnout,^5,7^ however the current study highlights that burnout is experienced in several healthcare disciplines beyond physicians alone. To this end, burnout scores were elevated among rehabilitation professionals (e.g., physical therapists, occupational therapists) who are less often included in healthcare burnout research. Research that has been conducted among rehabilitation professionals has been mixed. In one cross-sectional survey study of N=2813 physical therapists across the United States, 49.4% of respondents reported burnout.^40^ In contrast, another study of physical therapists operating in an orthopedics setting revealed burnout levels as low to moderate among respondents, however response rates were notably low (38.7%). Findings in the current study indicate that ehabilitation providers working in pediatric pain care experience levels of burnout commensurate with other healthcare disciplines (e.g., medicine), reinforcing the need for more multi-discipline burnout research.

Finally, client-related burnout was significantly lower than both personal and work-related burnout across all professions. While the reason for this difference cannot be clarified through the current study, several potential reasons exist. First, it is possible that survey responses were impacted by social desirability bias as the questions assessing client-related burnout may be particularly confronting for helping professionals who often see their profession as both a job and an identity or calling.^41^ Another possibility is that clients and families are *not* the source of provider burnout, however, the patient provider relationship has been previously identified as a source of stress for pain practitioners.^42^ Regardless, burnout remains a concern in the context of patient care as it has been documented to impact providers’ ability to empathize with families and build effective partnerships in care.^11,43^

### 4.2. Teamwork in pediatric pain care teams

Overall, providers reported positive perceptions of their teams’ functioning including their ability to communicate and share information with one another, support each other as a team, and be effective across several aspects of teamwork and coordination. Findings are consistent with previous research in healthcare teams, which have highlighted that providers value teamwork and believe that working as a team exceeds the potential of working as an individual provider.^44^ Moreover, these findings are promising as effective communication and information sharing has been tied to performance in teams across several different working domains.^45^ In healthcare specifically, teamwork has been examined across several different settings including emergency medicine, surgery, and intensive care with overall findings indicating that teamwork is related to performance outcomes (e.g., morbidity, adherence) and process outcomes (e.g., behaviors of speaking up during meetings, time to intervention).^46^ While mean scores across these facets of teamwork were high, it is important to note that variability did exist such that some providers reported significant concerns regarding their teams’ functioning, while others reported nearly perfect ratings. The variability in teamwork is also consistent with existing literature, as perceptions of teamwork can vary across time and professionals within a team.^47^ It is also possible that the current measures of team functioning were impacted by ceiling effects. To this end, it is plausible that small decrements in perceived team functioning may be indicative of more significant concerns given that external reporting of internal team dysfunction can be difficult due to factors such as in-group/out-group and social desirability biases. Thus, given the negative skew of these responses, any rating below the highest may indicate more substantial concern for team functioning.

#### 4.2.1. Relationship between burnout and team functioning

Correlation analyses between subscales of burnout and facets of team functioning indicated that provider perceptions of their teams’ functioning are related to their levels of burnout. The relationship between teamwork and burnout has not yet been assessed in pediatric care teams, however, these findings are consistent with previous research in adult pain care teams that identified team functioning as a factor that can impact individual provider experiences of burnout.^11^ In contrast, correlations between burnout and variables such as years of experience or patients seen per day were low, further supporting the need to look beyond individual demographic or clinical factors alone to understand provider experiences of burnout.^48^ While these factors may have indirect effects on teamwork and burnout (e.g., increased demand for billable hours decreases time for care coordination), they alone did not explain provider experiences. Finally, the K-means analysis meaningfully grouped providers, according to burnout subscales (personal, work-related, client-related burnout), into one of three groups: high levels of burnout, medium levels of burnout, and low levels of burnout. Subsequent groupwise comparisons between groups highlighted that while other demographic and clinical characteristics are similar across providers with low, moderate, and high burnout, perceptions of team functioning were significantly different between groups. These findings highlight the potential role of team functioning in provider burnout, highlighting teamwork as a factor that can exacerbate or mitigate the experience of burnout. By addressing teamwork, we may enhance clinical collaboration and reduce burnout among healthcare team members which may in turn improve collaboration between the healthcare team and patients/families.

### 4.3. Limitations & future research

The limitations of the current study warrant attention and prompt future investigation. First, as the current study was cross-sectional in nature, we cannot comment on causality between burnout and teamwork. While there does appear to be a linear relationship between burnout and perceptions of teamwork, it is unclear whether negative team experiences result in increased symptoms of burnout, or, if experiences of burnout negatively color provider perceptions of teamwork. Future research should be conducted longitudinally and within team cohorts to better elucidate the causality this relationship. To this end, given the anonymity of responses it is possible that multiple providers from the same program are represented and have some interdependent experiences, although this warrants further exploration. Qualitative methodologies may be useful for illuminating the relationship between provider burnout and teamwork including the specific drivers of teamwork that are most related to provider experiences of burnout (e.g., power differentials on teams, team hierarchy, shared decision-making) and whether experiences on a team are indeed shared and to what extent. Additionally, although we asked providers about their clinical hours and caseload specific to pediatric primary pain, we did not ask about other work and clinical commitments. To this end, we cannot distinguish if elevated burnout is due to pain related work alone. Future research should break this out more specifically to understand sources of burnout across clinical, administrative, teaching, and research commitments. Driven by the results of the K-means clustering analysis, we grouped providers into low, moderate, and high levels of burnout as they appear to represent distinct groups of providers with distinct experiences. However, since the CBI does not have established clinical cutoffs, a deeper understanding of the differences in experiences between these groups is needed to inform the development of targeted interventions for providers in different groups. Third, the current study examined burnout and team functioning from individual providers, limiting the perspective to a single person on the team. Given that researchers have demonstrated that perceptions of team functioning can vary across providers on a team,^47^ future research should target whole teams rather than individual providers to better understand how team functioning is perceived across all involved in the team. Observational research on team interactions may be insightful to achieving this aim. Finally, given the approach to recruitment that included listserv dissemination and snowball sampling, we do not know how many providers were made aware of the study and thus we cannot be sure of the representativeness of the current sample, which may have been impacted by self-selection bias.

## 5. Conclusion

This study was one of the first to examine burnout in pediatric pain care teams, expanding assessment of burnout to *all* providers who work on pain care teams (e.g., recreational therapists, physical therapists, social workers). Results highlight the prevalence of burnout among diverse pain care providers working as part of an integrated pain care team. Moreover, the study illustrates that, while teamwork is overall perceived positively by team members, variability in teamwork ratings exist and warrant attention. More importantly, findings suggest that perceptions of teamwork and experiences of burnout are related to one another. This underscores an important area for intervention in the effort to mitigate burnout in pediatric pain. Targeting the functioning of pain care teams may offer a new avenue through which we can mitigate provider burden, increase quality of life for pain care providers, and address burnout-driven provider turnover.

## Supplementary Material

SupplementalFileA

## Figures and Tables

**Table 1:** Provider & Clinic Characteristics.

Provider Demographic & Clinical Characteristics Across Professional domains						
	Behavioral Health	Medicine	Nursing	Rehab Provider	Other	Total Sample
n	53	48	21	63	10	195
Age (n (%))						
20–30	0 (0)	1 (2)	0 (0)	21 (33)	3 (30)	25 (13)
31–40	24 (45)	14 (29)	2 (10)	26 (41)	1 (10)	67 (34)
41–50	21 (40)	12 (25)	11 (52)	7 (11)	2 (20)	53 (27)
51–60	6 (11)	13 (27)	5 (24)	7 (11)	4 (40)	35 (18)
61–70	2 (4)	8 (17)	3 (14)	2 (3)	0 (0)	15 (8)
>71	0 (0)	0 (0)	0 (0)	0 (0)	0 (0)	0 (0)
Cultural Identity (n (%))						
African	0 (0)	0 (0)	1 (7)	3 (6)	0 (0)	4 (2)
East Asian	1 (2)	1 (2)	0 (0)	2 (4)	0 (0)	4 (2)
European	49 (94)	35 (76)	10 (67)	40 (78)	7 (88)	141 (81)
Hispanic/Latine	1 (2)	2 (4)	0 (0)	0 (0)	0 (0)	3 (2)
Indigenous	0 (0)	0 (0)	0 (0)	0 (0)	0 (0)	0 (0)
South Asian	0 (0)	5 (11)	1 (7)	2 (4)	0 (0)	8 (5)
Southeast Asian	0 (0)	1 (2)	1 (7)	0 (0)	0 (0)	2 (1)
West Central Asian	0 (0)	0 (0)	0 (0)	1 (2)	0 (0)	1 (1)
A Different Culture	1 (2)	2 (4)	2 (13)	5 (9)	1 (13)	11 (6)
Gender Identity						
Man	10 (18.9)	25 (52.1)	0 (0.0)	7 (11.3)	0 (0.0)	42 (21.6)
Woman	43 (81.1)	23 (47.9)	21 (100.0)	54 (87.1)	10 (100.0)	151 (77.8)
Nonbinary	0 (0.0)	0 (0.0)	0 (0.0)	1 (1.6)	0 (0.0)	1 (0.5)
Number of Patients Seen per Week with Primary Pain Concern (n (%))						
1–10	33 (62.3)	21 (43.8)	11 (52.4)	54 (85.7)	7 (70.0)	126 (64.6)
11–20	15 (28.3)	17 (35.4)	8 (38.1)	9 (14.3)	2 (20.0)	51 (26.2)
21–30	3 (5.7)	8 (16.7)	2 (9.5)	0 (0.0)	1 (10.0)	14 (7.2)
31–40	2 (3.8)	0 (0.0)	0 (0.0)	0 (0.0)	0 (0.0)	2 (1.0)
>40	0 (0.0)	2 (4.2)	0 (0.0)	0 (0.0)	0 (0.0)	2 (1.0)
Licensed Professional (% yes)	52 (98.1)	45 (95.7)	21 (100.0)	63 (100.0)	10 (100.0)	191 (98.5)
Years of Experience in Pediatric Pain (n (%))						
0–5	16 (30.2)	11 (23.4)	5 (23.8)	33 (52.4)	5 (50.0)	70 (36.1)
6–10	13 (24.5)	11 (23.4)	5 (23.8)	15 (23.8)	4 (40.0)	48 (24.7)
11–15	13 (24.5)	8 (17.0)	5 (23.8)	6 (9.5)	1 (10.0)	33 (17.0)
>15	11 (20.8)	17 (36.2)	6 (28.6)	9 (14.3)	0 (0.0)	43 (22.2)
Hours in Clinical Care per Week with Patients with Primary Pain (n (%))						
0–10	21 (39.6)	12 (26.1)	4 (19.0)	24 (38.1)	6 (60.0)	67 (34.7)
11–20	14 (26.4)	11 (23.9)	2 (9.5)	19 (30.2)	2 (20.0)	48 (24.9)
21–30	9 (17.0)	6 (13.0)	5 (23.8)	6 (9.5)	1 (10.0)	27 (14.0)
31–40	8 (15.1)	6 (13.0)	8 (38.1)	13 (20.6)	1 (10.0)	36 (18.7)
41–50	1 (1.9)	6 (13.0)	2 (9.5)	1 (1.6)	0 (0.0)	10 (5.2)
>50	0 (0.0)	5 (10.9)	0 (0.0)	0 (0.0)	0 (0.0)	5 (2.6)
Hours Spent in Care Coordination (mean (SD))	6.09 (5.61)	7.93 (5.56)	14.60 (10.67)	8.05 (6.00)	12.50 (11.11)	8.40 (7.16)
Clinical Setting (n (%))						
Urban	38 (74.5)	40 (88.9)	18 (85.7)	54 (87.1)	8 (88.9)	158 (84.0)
Suburban	12 (23.5)	5 (11.1)	3 (14.3)	8 (12.9)	0 (0.0)	28 (14.9)
Rural	1 (2.0)	0 (0.0)	0 (0.0)	0 (0.0)	1 (11.1)	2 (1.1)
Clinician Residence (n (%))						
North America	42 (79.2)	34 (70.8)	18 (85.7)	49 (77.8)	8 (80.0)	151 (77.4)
Europe	9 (17.0)	8 (16.7)	0 (0.0)	4 (6.3)	0 (0.0)	21 (10.8)
Australia	2 (3.8)	3 (6.2)	3 (14.3)	9 (14.3)	2 (20.0)	19 (9.7)
Somewhere Else	0 (0.0)	3 (6.2)	0 (0.0)	1 (1.6)	0 (0.0)	4 (2.1)

**Table 2:** Burnout Scores Across Professional Domains.

Burnout Scores Across Professional Domains							
	BehavioralHealth	Medicine	Nursing	RehabProvider	Other	p value	Total Sample
n	53	48	21	63	10		195
Personal Burnout (mean(SD))	36.79 (16.96)	39.76 (16.35)	42.26 (20.51)	42.00 (16.18)	39.17 (17.48)	0.533	39.91 (16.97)
Work-Related Burnout (mean(SD))	36.05 (17.17)	39.21 (16.66)	37.93 (14.22)	43.37 (18.92)	41.43 (23.22)	0.262	39.67 (17.75)
Client-Related Burnout (mean(SD))	23.19 (15.73)	27.08 (18.21)	19.64 (15.26)	29.70 (20.00)	18.33 (18.55)	0.078	25.62 (18.13)
Total Burnout (mean(SD))	32.22 (14.09)	35.55 (15.04)	33.52 (11.89)	38.62 (16.20)	33.42 (18.39)	0.225	35.31 (15.13)

Note. Established means come from the initial validation cohort which was comprised of human service workforce in Denmark as part of the PUMA study. Mean scores ranged from 29.5 to 44.7 (M=35.9) for personal burnout, 26.4–43.5 (M=33.0) for work-related burnout, and 19.7–41.2 (M=30.9) for client-related burnout.

**Table 3:** Team Functioning Scores.

Team Functioning Ratings Across Professional Domains							
	Behavioral Health	Medicine	Nursing	Rehab Provider	Other	p value	Total Sample
n	53	48	21	63	10		195
Communication & Sharing Information	25.68 (4.65)	27.21(4.64)	26.33 (5.71)	25.58 (5.41)	27.11 (5.40)	0.455	26.16 (5.05)
Mean Effectiveness	4.91 (1.10)	5.18 (1.06)	4.94 (1.23)	4.98 (1.02)	4.86 (1.11)	0.782	5.00 (1.07)
Mean Room for Improvement	3.38 (1.21)	3.08 (1.01)	2.99 (1.19)	3.14 (1.01)	3.42 (1.44)	0.566	3.19 (1.11)
Team Support	44.19 (7.27)	45.51 (7.53)	43.63 (7.97)	44.83 (8.51)	44.44 (6.56)	0.903	44.66 (7.74)

Note. Communication & Sharing Information scores range from 8 to 32; Mean Effectiveness scores range from 0 to 7; Mean Room for Improvement scores range from 0 to 7; Team Support scores range from 9 to 54.

**Table 4:** Item Means for Current Effectiveness & Room for Improvement.

	Mean Effectiveness	Mean Improvement
Being Ethical in our work	6.00	2.05
Establishing Treatment Goals	5.35	2.80
Working Together as a Team	5.30	3.03
Involving Families & Caregivers in Decisions	5.29	2.82
Providing Education to Families	5.28	3.03
Remaining Focused on Treatment Goals	5.24	2.93
Integrating New Information	5.12	3.51
Achieving Treatment Goals	5.08	3.06
Problem Solving Challenges	5.01	3.66
Adapting to Changes in Patient Status	4.98	3.16
Assisting Families with Problems	4.87	3.31
Dealing with Disagreements as a Team	4.77	3.21
Incorporating Divergent Perspectives	4.77	3.23
Overcoming Misunderstandings	4.65	3.36
Trying Innovative Strategies	4.64	3.63
Coordinating Activities	4.52	3.58
Using Team Meetings Productively	4.48	3.78

**Table 5:** Mean Burnout Scores Across Clustered Groups.

Cluster Centers Across Burnout Subscales			
	Personal Burnout	Work Related Burnout	Client Related Burnout
Low Burnout Symptoms	25.23	23.07	12.16
Moderate Burnout Symptoms	44.43	44.06	28.14
High Burnout Symptoms	61.33	65.85	49.74

**Table 6:** Groupwise comparisons of demographic and team functioning ratings across clustered burnout groups (low, moderate, high burnout).

Groupwise Comparisons Across Clustered Groups					
	Low Burnout	Moderate Burnout	High Burnout	p-value
n	74	89	32	
Professional Domain (n (%))				0.460
Medicine	18 (24.3%)	22 (24.7%)	8 (25.0%)	
Behavioral Health	26 (35.1%)	21 (23.6%)	6 (18.8%)	
Nursing	7 (9.5%)	12 (13.5%)	2 (6.2%)	
Rehabilitation	19 (25.7%)	29 (32.6%)	15 (46.9%)	
Different Domain	4 (5.4%)	5 (5.6%)	1 (3.1%)	
Age (n(%))			0.218	
20–30	6 (8.1%)	14 (15.7%)	5 (15.6%)	
31–40	23 (31.1%)	30 (33.7%)	14 (43.8%)	
41–50	19 (25.7%)	29 (32.6%)	5 (15.6%)	
51–60	18 (24.3%)	12 (13.5%)	5 (15.6%)	
61–70	8 (10.8%)	4 (4.5%)	3 (9.4%)	
Number of Patients Seen per Week with Primary Pain Concern (n (%))				0.371
1–10	46 (62.2%)	59 (66.3%)	21 (65.6%)	
11–20	25 (33.8%)	19 (21.3%)	7 (21.9%)	
21–30	2 (2.7%)	9 (10.1%)	3 (9.4%)	
31–40	1 (1.4%)	1 (1.1%)	0 (0.0%)	
>40	0 (0.0%)	1 (1.1%)	1 (3.1%)	
Team Type n (%)				
Outpatient Multidisciplinary	45 (60.8%)	61 (68.5%)	20 (62.5%)	
Intensive Interdisciplinary Pain Treatment	29 (39.2%)	28 (31.5%)	12 (37.5%)	
Licensed Professional (% yes)	71 (95.9%)	89 (100%)	32 (100%)	0.085
Years of Experience in Peds Pain (n (%))				0.059
0–5	29 (39.2%)	30 (34.1%)	11 (34.4%)	
6–10	11 (14.9%)	26 (29.5%)	11 (34.4%)	
11–15	10 (13.7%)	18 (20.5%)	5 (15.6%)	
>15	24 (32.4%)	14 (15.9%)	5 (15.6%)	
Hours in Clinical Care per Week with Patients with Primary Pain (n (%))				0.330
0–10	22 (30.1%)	34 (38.6%)	11 (34.4%)	
11–20	21 (28.8%)	20 (22.7%)	7 (21.9%)	
21–30	10 (13.7%)	11 (12.5%)	6 (18.8%)	
31–40	15 (20.5%)	17 (19.3%)	4 (12.5%)	
41–50	5 (6.8%)	4 (4.5%)	1 (3.1%)	
>50	0 (0.0%)	2 (2.3%)	3 (9.4%)	
Hours in Care Coordination (mean(SD))	8.54 (7.56%)	8.41 (7.56%)	8.06 (5.00%)	0.952
Clinic Setting (n (%))				0.291
Urban	56 (78.9%)	73 (85.9%)	29 (90.6%)	
Suburban	13 (18.3%)	12 (14.1%)	3 (9.4%)	
Rural	2 (2.8%)	0 (0.0%)	0 (0.0%)	
Clinician Residence (n (%))				n/a
North America	52 (70.3%)	74 (83.1%)	25 (78.1%)	
Europe	13 (17.6%)	7 (7.9%)	1 (3.1%)	
Australia	7 (9.5%)	6 (6.7%)	6 (18.8%)	
Caribbean Islands	0 (0.0%)	0 (0.0%)	0 (0.0%)	
Somewhere Else	2 (2.7%)	2 (2.2%)	0 (0.0%)	
Gender (n (%))				0.099
Man	22 (30.1%)	12 (13.5%)	8 (25.0%)	
Nonbinary	0 (0.0%)	1 (1.1%)	0 (0.0%)	
Woman	51 (69.9%)	76 (85.4%)	24 (75.0%)	
Mean Room for Improvement (mean(SD))	2.82 (1.07)^a^	3.39 (1.02)^b,c^	3.53 (1.24)^c^	0.002
Mean Effectiveness (mean (SD))	5.35 (1.00)^a^	4.89 (1.04)^b,c^	4.52 (1.11)^c^	0.001
Communication & Information Sharing (mean(SD))	28.23 (3.78)^a^	25.15 (5.29)^b,c^	24.19 (5.43)^c^	<0.001
Team Support (mean(SD))	47.97 (5.04)^a^	43.42 (8.47)^b,c^	40.36 (7.84)^c^	<0.001
Personal Burnout (mean (SD))	25.23 (8.83)^a^	44.43 (12.31)^b^	61.33 (11,84)^c^	<0.001
Work-Related Burnout (mean(SD))	23.07 (8.30)^a^	44.06 (8.90)^b^	65.85 (12.83)^c^	<0.001
Client-Related Burnout (mean(SD))	12.16 (8.63)^a^	28.14 (13.62)^b^	49.74 (17.42)^c^	<0.001

## Data Availability

All data are available upon reasonable request to the corresponding author.

## Perspective

Elevated burnout exists among pain care providers, and their burnout is related to their perceptions of their teams’ functioning. Providers with high burnout have more negative perceptions of their teams’ functioning compared to providers with low burnout. Teamwork processes may offer a novel and impactful target for future burnout interventions.

## Appendix A. Supporting information

Supplementary data associated with this article can be found in the online version at doi:10.1016/j.jpain.2026.106206.
