# Supplementary material for: Burnout and team functioning in pediatric pain care: A cross-sectional survey of multidisciplinary providers
Source: J Pain. Author manuscript; Available in PMC 2026 Jun 23. (PMC13286880; doi:10.1016/j.jpain.2026.106206)
Supplement: SupplementalFileA [file NIHMS2183235-supplement-SupplementalFileA.docx]

| Care Coordination Activities (n (%)) | | | | | | |
| --- | --- | --- | --- | --- | --- | --- |
|  | Behavioral Health | Medicine | Nursing | Rehabilitation | Other | Total Sample |
|  | n=53 | n=48 | n=21 | n=63 | n=10 | N=195 |
| Multidisciplinary rounds / Multispecialty team meeting | 48 (90.6%) | 42 (87.5%) | 18 (85.7%) | 59 (93.7%) | 8 (80.0%) | 175 (89.7%) |
| Team huddles (informal, curbside discussions) | 46 (86.8%) | 37 (77.1%) | 14 (66.7%) | 52 (82.5%) | 8 (80.0%) | 157 (80.5%) |
| 1:1 consultation (e.g., provide expertise to sports medicine clinic on specific client or topic) | 38 (71.7%) | 29 (60.4%) | 9 (42.9%) | 36 (57.1%) | 3 (30.0%) | 115 (59.0%) |
| Collateral contacts (e.g., phone call, messaging, email) within your institution (e.g., psychiatry, rheumatology, child life, nursing) | 49 (92.5%) | 43 (89.6%) | 19 (90.5%) | 53 (84.1%) | 8 (80.0%) | 172 (88.2%) |
| Collateral contacts (e.g., phone call, messaging, email) outside of your institution (e.g., teachers, coaches, choir director) | 44 (83.0%) | 27 (56.2%) | 13 (61.9%) | 48 (76.2%) | 7 (70.0%) | 139 (71.3%) |
| Morning rounds with immediate team (within discipline or specialty service) | 15 (28.3%) | 23 (47.9%) | 6 (28.6%) | 20 (31.7%) | 6 (60.0%) | 70 (35.9%) |
| Multidisciplinary patient care meeting/patient care conference (with patient) | 41 (77.4%) | 42 (87.5%) | 17 (81.0%) | 54 (85.7%) | 7 (70.0%) | 161 (82.6%) |
